# Self-reported flares are predictors of radiographic progression in rheumatoid arthritis patients in 28-joint disease activity score remission: a 24-month observational study

**DOI:** 10.1186/s13075-016-0986-1

**Published:** 2016-04-14

**Authors:** Francesca Ometto, Bernd Raffeiner, Livio Bernardi, Costantino Bostsios, Nicola Veronese, Leonardo Punzi, Andrea Doria

**Affiliations:** Rheumatology Unit, Department of Medicine, University of Padova, Via Giustiniani, 2, 35128 Padova, Italy; Rheumatology Unit, Department of Medicine, Bolzano General Hospital, Via Lorenz Bohler, 5, 39100 Bolzano, Italy; Department of Medicine, University of Padova, Via Giustiniani, 2, 35128 Padova, Italy; Institute of clinical Research and Education in Medicine (IREM), Padova, Italy

**Keywords:** Flares, Self-reported flares, Rheumatoid arthritis, DAS28 remission, Radiographic progression, Biologic treatment

## Abstract

**Background:**

Disease flares are common in rheumatoid arthritis (RA) and are related to structural damage. However, few data on the impact of flares reported by patients on radiographic progression are available. Our aim was to investigate whether overall flares (OF), self-reported flares (SRF) and short flares assessed at the visit (SF) predict radiographic progression in RA patients in DAS28 (28-joint disease activity score) remission.

**Methods:**

We reviewed the records of RA patients included in our database. We considered all patients who had a period of at least 24 months in remission (DAS28 < 2.6), stable biologic and synthetic disease-modifying anti-rheumatic drug treatment, no missing follow-up visits and hands and feet radiographs at the start and at the end of the 24-month follow up. Radiographic progression was considered as an increase in the van der Heijde modified total Sharp score >0. Patients were assessed every 3 months and flares were recorded. We defined SRF as any worsening of the disease reported by patients occurring in the time between visits and SF as an increase in DAS28 ≥ 2.6 or >0.6 from the previous visit assessed by the physician in one isolated visit. The impact of SRF, SF and OF on radiographic progression was assessed through multivariate regression analysis.

**Results:**

One hundred forty-nine patients were included. The median number (interquartile range) of OF was 1.00/year (0.50; 1.38), of SRF was 0.50/year (0.14; 1.00), and of SF was 0.34/year (0; 0.50). Eighteen patients (12.1 %) experienced a progression of radiographic damage. OF and SRF were significant predictors of radiographic progression: OR 3.27, 95 % CI 1.30, 8.22 and OR 3.63, 95 % CI 1.16, 11.36, respectively.

**Conclusions:**

OF and SRF are predictors of structural damage. Flares assessed at the visit, SF, do not impact on radiographic progression as they might underestimate the actual number of flares.

**Electronic supplementary material:**

The online version of this article (doi:10.1186/s13075-016-0986-1) contains supplementary material, which is available to authorized users.

## Background

Rheumatoid arthritis (RA) is a chronic inflammatory disease leading to joint disruption and eventually, disability. Treatment with biologic drugs has made clinical remission an achievable target. Nevertheless, periodic fluctuations of disease activity are common in RA [1] and occur also in patients in remission [2].

Although several efforts have been undertaken by international initiatives, no validated definition of flare is available. The Outcome Measures in Rheumatology Clinical Trials group has recently proposed a core domain set to measure RA flare [3], and the French Strategy of Treatment in Patients with Rheumatoid Arthritis Group has developed a patient self-assessed questionnaire, FLARE, to identify fluctuations of disease activity that occur in the time between visits [4].

Some considerable interest has arisen on what impact flares have on the success of the treatment and on structural damage. Temporary variations in disease activity have already been related to radiographic evidence of progression [5–7] and disease flares at the time of the visit are proved to be associated with disability [6, 7].

To date there is limited knowledge about flares reported by patients and no information on the impact of these flares on structural damage. The frequency and duration of flares reported by patients have been investigated in an observational study by means of a questionnaire: 99 % of patients, including those in remission, reported flares in the 6 months before the visit over 3 years of follow up [8].

The aim of our study was to investigate whether flares predict radiographic evidence of progression in RA patients in 28-joint disease activity score (DAS28) remission, examining flares reported by patients, self-reported flares (SRF), and short flares (SF) defined according to the DAS28 at the time of the visit.

## Methods

### Patients

We conducted a retrospective observational study in the outpatient clinic of the Rheumatology Unit of Padova University Hospital. All participants provided written informed consent before inclusion in the study. The study was carried out in accordance with the ethical standards of the Declaration of Helsinki (1983) and was approved by the Ethics Committee for the clinical trials of the province of Padova.

All patients had a diagnosis of RA according to the American College of Rheumatology 1987 classification criteria [9] and had started treatment with a subcutaneous anti-tumour necrosis factor α agent between January 2009 and October 2012. We reviewed the clinical records of our patients and we included in the study those who had had a period of at least 24 months in remission and stable biologic and disease-modifying anti-rheumatic drug (DMARD) treatment.

Remission was defined as a DAS28 < 2.6, calculated using C-reactive protein (CRP) [10]. Patients who were out of remission on one isolated visit (DAS28 ≥ 2.6) within the 24-month period were maintained in the study; patients with a DAS28 ≥ 2.6 on two consecutive visits were excluded.

We considered patients on full-dose and on low-dose biologic treatment. In our clinical practice, patients who maintained remission for at least 6 months on a full-dose biologic drug underwent dose reduction and continued on low dose until remission was maintained. To ensure homogeneous follow-up and treatment strategies, only patients on adalimumab (ADA) and etanercept (ETA) were included. Full-dose treatments were ETA 25 mg twice weekly and ADA 40 mg every 2 weeks; low-dose treatments were ETA 25 mg weekly and ADA 40 mg every 3 weeks.

Concomitant DMARDs were methotrexate (MTX) (10–15 mg weekly) or leflunomide (LFN) (10–20 mg daily). Prednisone (PDN) could be used at a dose of ≤5 mg daily.

Data collected were: age, sex, disease duration at the start of the current biologic treatment, seropositivity (positive anti-citrullinated peptides or positive rheumatoid factor), smoking status, previous anti-TNFα treatments and biologic, DMARD and corticosteroid dose. Patients were treated with a treat-to-target strategy and assessed every 3 months. At each follow-up visit, the DAS28 and the Health Assessment Questionnaire (HAQ) were recorded.

Radiographs of the hands and feet were performed every year in clinical practice. We collected radiographs at the start and at the end of the 24-month follow up and the van der Heijde modified total Sharp score (TSS) was calculated by one radiologist. Mean TSS progression per year before baseline was also calculated. Radiographic progression was considered as evidence of an increase in the TSS >0 at the 24-month follow up.

### Flares

Flares were classified as SRF and SF. SRF were defined as any worsening of the disease reported by patients, occurring in the time between visits. At every follow-up visit patients were asked if they had experienced a worsening of the symptoms related to RA. Any symptom attributed by the patient to RA (i.e., joint pain, joint swelling, stiffness and constitutional symptoms) was considered. SF were defined according to the DAS28, being a DAS28 ≥ 2.6 or an increase in the DAS28 > 0.6 from the previous visit assessed by the physician at one isolated visit. The opinion of the patient on whether he or she was experiencing a flare at the time of the visit was not collected because this was obtained in an assessment of the patient’s global health measured on a visual analog scale (patient-VAS) as part of the DAS28. Flare duration (as reported by the patient at the following visit), symptoms and treatment changes were recorded. OF was defined as the sum of SF and SRF.

Exclusion criteria were: missing follow-up visits, missing radiographs or missing data during the 24-month follow up; concomitant musculoskeletal conditions (e.g., fibromyalgia, osteoarthritis) and unreliability in reporting flares (i.e., patients with cognitive impairment or lack of proficiency in the Italian language).

### Statistical analysis

Data are presented in all patients and according to the occurrence of radiographic progression at the 24-month follow up. Normal distributions of continuous variables were tested using the Shapiro-Wilk test and, if normality was satisfied, the data were shown as means ± standard deviations (SD) and compared using the unpaired *t* test. Variables with a non-normal distribution were presented as medians with the corresponding interquartile range (IQR) and compared using the Mann-Whitney test. Quantitative measures were compared using t chi-square test or Fisher’s exact test. Flares are presented as the mean number of flares per year.

Multivariate analysis was run to assess the potential of the numbers of OF, SRF and SF (independent variables) to predict radiographic progression (dependent variable). The impact of OF (model I), SRF (model II) and SF number (model III) was assessed separately. The predictors included in the final model were all variables with a *p*-value <0.20 in the univariate analyses as reported in Additional file 1: Table S1.

Collinearity was assessed by the variance inflation factor (VIF), adopting a cut off of VIF = 2 as an exclusion criterion. Variables excluded from the multivariate analysis because of collinearity were the same in the three models: HAQ at 24 months collinear with baseline HAQ (model I VIF = 13.34, model II VIF = 12.77; model III VIF = 13.38) and patient-VAS at 24 months collinear with change in the patient-VAS at 24 months (model I VIF = 3.08, model II VIF = 2.98, model III VIF = 3.01). The results of univariate and multivariate logistic regression analysis are presented as the odds ratio (OR) with the corresponding 95 % confidence interval (CI). All analyses were performed using SPSS version 22.0.

## Results

Among 435 patients who started ADA or ETA, 323 fulfilled the inclusion criteria, i.e., they had a period of 24 months in remission, and stable biologic and DMARD treatment. One hundred thirty-seven patients were excluded because of missing follow-up visits, missing radiographs or missing data during the 24-month follow up. Thirty-seven patients were excluded because of concomitant musculoskeletal comorbidities (7 patients with fibromyalgia and 9 with osteoarthritis) or because they were not reliable in reporting flares (13 with cognitive impairment and 3 patients with lack of proficiency in the Italian language), leaving 149 patients (56 on ADA and 93 on ETA) eligible for the analysis. The characteristics of patients and treatments at baseline are reported in Table 1. HAQ, DAS28 and DAS28 components at baseline and at the 24-month follow up are also detailed.

**Table 1:** Demographics and clinical variables according to radiographic progression at the 24-month follow up

	All patients	No radiographic progression	Radiographic progression	*P* value^a^
Number	149	131	18	
Age, years, mean (SD)	58.15 (12.58)	57.60 (12.71)	62.22 (11.01)	0.11
Female, *n* (%)	123 (82.6)	108 (82.4)	15 (83.3)	0.61
Disease duration, years, median (IQR)	13.00 (8.00; 21.00)	13.00 (8.00; 20.50)	13.50 (8.50; 30.75)	0.41
Previous anti-TNFα failures, *n* (%)	30 (20.1)	26 (19.9)	4 (22.2)	0.51
Positive ACPA and/or RF, *n* (%)	76 (51.0)	65 (49.6)	11 (61.1)	0.36
Smokers or ex-smokers, *n* (%)	39 (26.2)	35 (26.7)	4 (22.2)	0.47
TSS progression per year before baseline, median (IQR)	8.00 (5.00; 11.63)	7.32 (5.00; 11.10)	10.25 (7.38; 15.50)	0.02
ADA treatments, *n* (%)	56 (37.6)	48 (36.6)	8 (44.4)	0.59
ETA treatments, *n* (%)	93 (62.4)	83 (63.4)	10 (55.6)	0.59
Low dose biologic, *n* (%)	103 (69.1)	89 (67.9)	14 (77.8)	0.40
Concurrent DMARD use, *n* (%)	76 (51.0)	66 (50.4)	10 (55.6)	0.68
PDN daily dose, mg, median (IQR)	1.00 (0.74; 2.00)	1.00 (0.60; 2.00)	1.50 (0.75; 2.00)	0.99
TSS progression at the 24-month follow up, median (IQR)	0 (0)	0 (0)	2.00 (2.00; 14.00)	<0.01
Baseline				
HAQ, mean (SD)	0.74 (0.40)	0.73 (0.41)	0.87 (0.25)	0.08
DAS28, median (IQR)	2.21 (1.92; 2.38)	2.24 (1.85; 2.38)	2.05 (2.05; 2.38)	0.53
CRP, mg/L, median (IQR)	3.00 (1.00; 4.00)	3.00 (1.00; 4.00)	1.00 (1.00; 4.00)	0.24
TJC, median (IQR)	1 (0; 2)	1 (0; 2)	1 (1; 1)	0.70
SJC, median (IQR)	0 (0)	0 (0)	0 (0)	–
Patient-VAS, mm, median (IQR)	20 (15; 20)	20 (10; 30)	20 (0)	0.23
Follow up, 24 months				
HAQ, median (IQR)	0.74 (0.39)	0.73 (0.40)	0.85 (0.23)	0.18
DAS28, median (IQR)	2.16 (1.80; 2.43)	2.18 (1.80; 2.63)	1.85 (1.82; 2.42)	0.52
CRP, mg/L, median (IQR)	2.00 (1.00; 3.00)	2.00 (1.00; 3.00)	2.00 (2.00; 3.00)	0.29
TJC, median (IQR)	1 (0; 2)	1 (0; 2)	0 (0; 1)	0.24
SJC, median (IQR)	0 (0)	0 (0)	0 (0)	–
Patient-VAS, mm, median (IQR)	20 (10; 30)	20 (10; 30)	33 (28; 35)	0.01
Change between baseline and 24-month follow up				
HAQ, median (IQR)	0 (−0.10; 0.07)	0 (−0.11; 0.07)	0.01 (−0.05; 0.05)	0.56
DAS28, mean (SD)	−0.13 (0.55)	0 (0.58)	−0.02 (0.30)	0.63
CRP, mg/L, median (IQR)	0 (−2.00; −2.00)	0 (−3.00; 2.00)	1.00 (−1.00; 1.00)	0.11
TJC, median (IQR)	0 (−1; −1)	0 (−1; −1)	−1 (−1; 0)	0.28
SJC, median (IQR)	0 (0)	0 (0)	0 (0)	–
Patient-VAS, mm, median (IQR)	5 (−5; 10)	0 (−10; 10)	13 (9; 15)	<0.01

*SD* standard deviation, *IQR* interquartile range, *TNFα* tumor necrosis factor-α, *ACPA* anti-citrullinated peptides, *RF* rheumatoid factor, *TSS* total Sharp score, *ADA* adalimumab, *ETA* etanercept, *DMARD* disease-modifying anti-rheumatic drug, *PDN* prednisone, *HAQ* Health Assessment Questionnaire, *DAS28* disease activity score in 28 joints, *CRP* C-reactive protein, *TJC* tender joint count in 28 joints, *SJC* swollen joint count in 28 joints, *patient-VAS* patient’s global health measured on a visual analog scale. ^a^Unpaired *t* test was used to compare variables with a normal distribution, Mann-Whitney test was used to compare variables with a non-normal distribution. Quantitative measures were compared using the chi-square test or Fisher’s exact test

There were 46 patients (30.9 %) on full-dose biologic agents and 103 (69.1 %) on low-dose agents, with 34 (60.7 %) on low-dose ADA and 69 (74.2 %) on low-dose ETA. A concomitant DMARD was used by 51 % of patients, with 50 patients on MTX and 26 on LFN. The median PDN daily dose was 1 mg daily (0.74; 2.00). At baseline median DAS28 was 2.21 (1.92; 2.38) and mean HAQ score was 0.75 ± 0.32; the DAS28 and HAQ score were stable over the 24-month follow up (Table 1).

In the 149 patients included in the study, there were 288 OF, 184 SRF, and 104 SF recorded over the 24-month follow up; 25 patients experienced no flares. The median number of OF was 1.00/year (0.50; 1.38), of SRF was 0.50/year (0.14; 1.00), and of SF was 0.34/year (0; 0.50) (Table 2). The mean duration of OF, as reported by the patients, was 12.59 ± 5.36 days (12.87 ± 5.49 for SRF and 13.04 ± 5.16 for SF, *p* = 0.77). No patients experienced flares lasting more than 30 days. Among patients with SRF, 84.8 % (156/184) reported joint swelling or tenderness, 65.8 % (123/187) reported stiffness, and 40.6 % (76/187) reported constitutional symptoms (i.e., fatigue or fever).

**Table 2:** Numbers of flares according to radiographic evidence of progression at 24-month follow up

	All patients	No radiographic progression	Radiographic progression	*P* value^a^
Number	149	131	18	
OF, number/year, median (IQR)	1.00 (0.50; 1.38)	0.98 (0.50; 1.00)	1.50 (1.00; 1.50)	<0.01
SRF, number/year, median (IQR)	0.50 (0.14; 1.00)	0.50 (0.10; 1.00)	1.00 (0.50; 1.00)	0.01
SF, number/year, median (IQR)	0.34 (0; 0.50)	0.24 (0; 0.50)	0.50 (0.24; 0.63)	0.08

*IQR* interquartile range, *OF* overall flares, *SRF* self-reported flares, *SF* short flares. ^a^The Mann-Whitney test was used to calculate *p* values

Temporary changes (lasting ≤30 days) in treatment were undertaken in most patients during flares; this was so in 266/288 patients (92.4 %) with OF, 172/184 patients (93.5 %) with SRF and 94/104 patients (90.4 %) with SF, *p* = 0.34. PDN dose was increased (up to 10 mg/day) in 173/288 patients (60.1 %) with OF, 115/184 patients (62.5 %) with SRF and 58/104 patients (55.8 %) with SF, *p* = 0.26. Non-steroidal anti-inflammatory drugs (NSAIDs) were used in 168/288 patients (58.3 %) with OF, 101/184 patients (54.9 %) with SRF and 67/104 patients (64.4 %) with SF, *p* = 0.11; intra-articular injections were used in 7/288 patients (2.4 %) with SF. The median DAS28 at the time of the flare in patients with SF was 3.22 (3.01; 3.87). On analysis of correlation between patient-VAS at the end of follow up and the number of OF the *R* value was 0.35, *p* < 0.01; for correlation between patient-VAS and the number of SRF the *R* value was 0.34, *p* < 0.01.

### Radiographic progression

There were 18 patients (12.1 %) with radiographic evidence of progression of damage (TSS >0) at the 24-month follow up; the median change in the TSS was 2, ranging from 1 to 14. Clinical variables and treatments taken by patients according to radiographic progression are reported in Table 1. The baseline HAQ score was higher in patients who had structural damage compared with those who did not (0.87 ± 0.25 and 0.73 ± 0.41, respectively, *p* = 0.05) and there was also a trend towards a higher HAQ score in these patients at the end of the 24-month follow up (Table 1). Progression in the TSS per year before baseline was significantly higher in patients who experienced radiographic progression compared with those who did not, with 10.25 (7.38; 15.50) vs 7.32 (5.00; 11.10), *p* = 0.02. Patient-VAS at the 24-month follow up was significantly higher in those with radiographic progression; likewise, the change in patient-VAS from baseline to the 24-month follow up was higher in the latter group, with 13 (9; 15) vs 0 (−10; 10), < 0.01 (Table 1).

The numbers of OF and SRF were significantly higher in patients with radiographic progression compared with those without radiographic progression, with 1.50 (1.00; 1.50) vs 0.98 (0.50; 1.00), *p* < 0.01 and 1.00 (0.50; 1.00) vs 0.50 (0.10; 1.00), *p* = 0.01, for OF and SF, respectively (Table 2). The number of SF was also higher in patients with radiographic progression, although not significantly, with 0.50 (0.24; 0.63) vs 0.24 (0; 0.50), *p* = 0.08 (Table 2).

The predictors of radiographic progression were tested by univariate regression analysis (Additional file 1: Table S1) and accordingly, covariates were selected for multivariate regression models. OF and SRF were independent predictors of radiographic progression in model I and II, respectively (Table 3), with OR of 3.27, 95 % CI 1.30, 8.22, *p* = 0.01 and 3.63, 95 % CI 1.16, 11.36, *p* = 0.03; whereas SF were not significant predictors of radiographic progression (OR 2.78, 95 % CI 0.70, 11.10, *p* = 0.15). In all three regression models, a unit increase in the baseline HAQ increased radiographic progression five-fold and patient-VAS change two-fold (Table 3). A higher TSS for progression per year before baseline was also an independent predictor of radiographic progression in all models (Table 3).

**Table 3:** Risk of radiographic progression: multivariate regression analysis

		Model I		Model II		Model III	
		Overall flares		Self-reported flares		Short flares	
		OR (95 % CI)	*P* value	OR (95 % CI)	*P* value	OR (95 % CI)	*P* value
Number		149		149		149	
Age	(per 10-year increase)	1.10 (0.67, 1.82)	0.70	1.16 (0.71, 1.89)	0.55	1.09 (0.67, 1.78)	0.72
TSS progression per year before baseline	(per unit)	1.12 (1.03, 1.23)	0.01	1.02 (1.02, 1.21)	0.02	1.10 (1.01, 1.20)	0.02
Baseline HAQ	(per unit)	5.32 (1.05, 26.99)	0.04	5.07 (1.08, 23.79)	0.04	4.66 (0.98, 22.25)	0.05
CRP change at 24 months	(per increasing quartile)	1.60 (0.87, 2.92)	0.13	1.66 (0.92, 3.01)	0.09	1.65 (0.92, 2.97)	0.09
Patient-VAS change at 24 months	(per 10-unit increase)	2.08 (1.14, 3.81)	0.02	2.06 (1.14, 3.72)	0.02	2.28 (1.29, 4.01)	0.04
OF	(per unit)	3.27 (1.30, 8.22)	0.01	–	–	–	–
SRF	(per unit)	–	–	3.63 (1.16, 11.36)	0.03	–	–
SF	(per unit)	–	–	–	–	2.78 (0.70, 11.10)	0.15

*OF* overall flares, *SRF* self-reported flares, *SF* short flares, *TSS* total Sharp score, *HAQ* health assessment questionnaire, *CRP* C-reactive protein, *Patient-VAS* patient’s global health measured on a visual analog scale

## Discussion

Our study investigates the impact of flares on structural damage in a clinical practice setting. Disease relapses at the time of the visit have already been found to be associated with radiographic progression [5–7], but to the best of our knowledge this is the first study that specifically considers flares reported by patients.

Previous studies proving the association between disease fluctuations and structural damage included patients with higher disease activity and adopted different definitions of flare. Welsing et al. [5] considered disease fluctuations as the standard deviation of the mean changes in the DAS (in 44 joints); Lillegraven et al. [6] considered the number of visits in remission and Markusse et al. [7] defined flares according to a DAS ≥2.4, which corresponds to moderate disease activity.

In our study, the flare definition included two different approaches, one objective definition based on the DAS28, the other relying on patient experience. Flares reported by patients, SRF, were found to independently predict radiographic progression; by contrast, flares assessed by the physician at the visit, SF, did not.

We defined SF according to the DAS28. The most discriminating and valid DAS28-based definition of flare has already been reported as an increase in DAS28 > 1.2 or >0.6 only if the current DAS28 is ≥3.2 [11]. We deemed this criterion not suitable for patients in remission, as it does not take into account the loss of remission, i.e., DAS28 ≥ 2.6. On the other hand, defining flare just as the loss of remission was shown to be non-specific [11]. To increase the sensitivity in the detection of mild worsening of disease activity, we also considered a change in the DAS28 > 0.6 from the previous visit as a criterion for SF, which is the DAS28 threshold in the response criteria of the European League Against Rheumatism (EULAR) [12].

SRF was defined as the worsening of any symptom attributed to RA by the patient, and no threshold for duration of symptoms or symptom severity was required. Although patients often describe a wide range of symptoms that are sometimes not related to RA in consultation with the rheumatologist [13], it has been shown that symptoms reported by patients with RA can complement the physician’s opinion, especially for systemic manifestations [14]. This study proves that the opinion of patients is needed to fully assess disease activity, as patients can identify significant worsening of it. SRF are also affected by memory bias. We chose a 3-month interval for recalling flares, as it has been suggested as the optimum time interval for detecting them [4] and it is a suitable interval in the follow up of RA. Despite the fact that this definition of SRF may be regarded as flawed, in our study SRF were independently associated with structural damage, supporting the evidence that patients can identify relevant disease relapses and active synovitis.

No differences were observed in the duration of SF and SRF, or in treatment administered. The greater impact of SRF on structural damage was probably due to a better estimate of the number of flares provided by SRF compared with SF. In fact, SF are identified on clinical assessment in one single visit and might underestimate disease activity over time.

A higher mean TSS progression per year before baseline and a higher HAQ at baseline were found to be predictors of radiographic progression. These indices are markers of a more severe and erosive arthritis thus conceiving the idea that disease severity is more likely associated with persistent disease activity and consequently with structural damage. An increase in patient-VAS over the study period was also significantly associated with radiographic evidence of disease progression, but it is probably associated with frequent relapses experienced by the patient rather than radiographic progression.

Treatment does not affect structural damage or the number of flares. No differences were observed in the number of SF, SRF or OF in relation to low- or full-dose biologic agents, or to concomitant DMARD treatment. This result is consistent with the treat-to-target treatment strategy and endorses the observation that the disease can be fully controlled, even on low-dose biologic agents, if patients are closely assessed [15]. This study provides further evidence that flares are common in RA patients even those in DAS28 remission [2]: considering SF and SRF together, patients had a median of 1 flare per year and SRF were almost two-fold more frequent than SF.

Although our study was limited to patients in remission and the definition of flare was different to definitions proposed by other authors, the overall number of flares that we observed was higher than reported in other studies [7, 16, 17]. Also, SRF appear to be more frequent compared with a previous study by Bykerk on flares reported by patients: 30 % of patients in remission reported ≥1 flare in the 6 months before the visit over a 3-year follow up compared with 83.2 % (124/149) of our patients, who reported ≥1 flare in the 3 months before the visit over a 2-year follow up [8]. This difference could be partly explained by more recall bias in the study by Bykerk, in which data on flares were collected every 6 months rather than every 3 months as in our study. Further, while we questioned patients at the time of the visit about flares, patients in the study by Bykerk were administered a questionnaire, which might have reduced patient compliance in providing the required information [8].

The major limitation of the study is its retrospective nature; a prospective study could improve the detection of flares, especially of SRF, through training patients to better identify the flares and symptoms that are associated with structural damage. Another limitation of our analysis is the small number of patients who achieved the outcome: only 12.1 % (18/149) of patients had radiographic evidence of disease progression, despite our definition of progression being a change in the TSS >0, instead of >0.5 as in previous studies [5–7]. This result was largely expected because the study was aimed at identifying inadequate disease control and minimal structural damage in patients in DAS28 remission during treatment with biologic agents. Nevertheless, OF and SRF proved to be strongly associated with radiographic progression (*p* = 0.01 and 0.03, respectively). Further, as there are no available validated definitions of patient-reported flares or of flares in patients in remission, the definitions we adopted were based on previous studies.

On the other hand, our study has some strengths as it was conducted in a monocentric cohort of patients with RA, which results in high homogeneity of the data collected, clinical assessments and treatment decisions. Further, a complete follow up of our patients was ensured by the fact that in our country, follow-up visits are mandatory for the prescription of the biological treatment.

## Conclusions

Flares are a common experience in patients with RA who are in DAS28 remission, and most of the flares are SRF occurring in the time between visits. OF and SRF impact on structural damage in patients with RA in DAS28 remission; by contrast, SF, assessed at the visit, do not, as they underestimate the actual number of flares. A definition of flare with thresholds for meaningful changes in RA symptoms is needed, in order to identify patients prone to developing structural damage, who might benefit from a treatment change despite their apparent remission state.

## Additional file

Additional file 1: Table S1. Risk of radiographic progression, results of univariate regression analysis. (PDF 75 kb)

## Abbreviations

ADA: adalimumab
CI: confidence interval
CRP: C-reactive protein
ETA: etanercept
DAS: disease activity score
DAS28: 28-joint disease activity score
DMARD: disease-modifying anti-rheumatic drug
HAQ: Health Assessment Questionnaire
IQR: interquartile range
LFN: leflunomide
MTX: methotrexate
OF: overall flares
OR: odds ratio
PDN: prednisone
RA: rheumatoid arthritis
SD: standard deviation
SF: short flares
SRF: self-reported flares
TSS: van der Heijde modified total Sharp score
VAS: visual analog scale
VIF: variance inflation factor
