# Erratum to: Self-reported flares are predictors of radiographic progression in rheumatoid arthritis patients in 28-joint disease activity score remission: a 24-month observational study

**DOI:** 10.1186/s13075-016-1019-9

**Published:** 2016-05-23

**Authors:** Francesca Ometto, Bernd Raffeiner, Livio Bernardi, Costantino Botsios, Nicola Veronese, Leonardo Punzi, Andrea Doria

**Affiliations:** Rheumatology Unit, Department of Medicine, University of Padova, Via Giustiniani, 2, Padova, 35128 Italy; Rheumatology Unit, Department of Medicine, Bolzano General Hospital, Via Lorenz Bohler, 5, Bolzano, 39100 Italy; Department of Medicine, University of Padova, Via Giustiniani, 2, Padova, 35128 Italy; Institute of clinical Research and Education in Medicine (IREM), Padova, Italy

Unfortunately, after publication of this article [1], it was noticed that the author name Constantino Botsios was incorrectly spelled during the production process. The corrected name can be seen in the author list above and the original article has also been updated to reflect this.
