# Supplementary material for: Self-reported flares are predictors of radiographic progression in rheumatoid arthritis patients in 28-joint disease activity score remission: a 24-month observational study
Source: Arthritis Res Ther. 2016 Apr 14;18:89. doi: 10.1186/s13075-016-0986-1 (PMC4831166; doi:10.1186/s13075-016-0986-1)
Supplement: Additional file 1: Table S1. — Risk of radiographic progression, results of univariate regression analysis. (PDF 75 kb) [file 13075_2016_986_MOESM1_ESM.pdf]

Table S1. Risk of radiographic progression: univariate regression analysis.

		OR (95% CI)	p value
Age	(per 10-years increase)	1.37 (0.90-2.09)	0.15
Males		1.07 (0.28-3.98)	0.93
Disease duration	(per 5-years increase)	1.02 (0.95-1.09)	0.67
Previous anti-TNF α failures		0.87 (0.26-2.85)	0.87
Positive ACPA and/or RF		1.60 (0.58-4.37)	0.36
Smokers or ex-smokers		1.28 (0.39-4.14)	0.69
TSS progression per year before baseline	(per unit)	1.07 (1.00-1.14)	0.06
Etanercept		0.85 (0.65-1.26)	0.67
Low dose biologic		0.61 (0.19-1.95)	0.40
Concurrent DMARD use		0.81 (0.30-2.19)	0.68
Mean PDN daily dose	(per unit)	0.92 (0.59-1.43)	0.70
**Baseline**			
HAQ	(per unit)	2.39 (0.71-8.13)	0.16
DAS28	(per unit)	0.75 (0.24-2.33)	0.62
CRP	(per increasing quartile)	0.79 (0.47-1.30)	0.35
TJC	(per unit)	0.75 (0.38-1.49)	0.42
SJC	(per unit)	-	-
Patient-VAS	(per 10-units increase)	1.03 (0.96-1.09)	0.42
**24-Month Follow-up**			
HAQ	(per unit)	2.17 (0.61-7.71)	0.20
DAS28	(per unit)	0.69 (0.22-2.15)	0.52
CRP	(per increasing quartile)	1.01 (0.64-1.61)	0.96
TJC	(per unit)	1.33 (0.46-6.72)	0.73
SJC	(per unit)	-	-
Patient-VAS	(per 10-units increase)	3.10 (1.46-6.59)	0.01
**Δ (Baseline - 24-Month Follow –up)**			
HAQ	(per unit)	1.30 (0.02-93.39)	0.90
DAS28	(per unit)	0.94 (0.38-2.3)	0.89
CRP	(per increasing quartile)	1.46 (0.92-2.35)	0.11
TJC	(per unit)	2.27 (0.73-0.70)	0.34
SJC	(per unit)	-	-
Patient-VAS	(per 10-units increase)	1.79 (1.17-2.74)	0.01
OF	(per unit)	3.11 (1.43-6.77)	0.01
SRF	(per unit)	3.43 (1.28-9.21)	0.02
SF	(per unit)	2.92 (0.83-10.19)	0.09

TNFα: tumour-necrosis-factor-α, ACPA: anti-citrullinated peptides; RF: rheumatoid factor, TSS: Total-Sharp Score, ADA: adalimumab, ETA: etanercept, DMARD: disease-modifying anti-rheumatic drug, PDN: prednisone, HAQ: health assessment questionnaire, DAS28: disease activity index - 28 joints, TJC: tender joint count on 28 joints, SJC: swollen joint count on 28 joints, patient-VAS: patient's global health measured on a Visual Analogic Scale, OF: overall flares, SRF: self-reported flares, SF: short flares.
